# Studies in two allopatric populations of *Hypostomus affinis* (Steindachner, 1877): the role of mapping the ribosomal genes to understand the chromosome evolution of the group

**DOI:** 10.3897/CompCytogen.v12i1.22052

**Published:** 2018-01-09

**Authors:** Karina de Oliveira Brandão, Dinaíza Abadia Rocha-Reis, Caroline Garcia, Rubens Pazza, Lurdes Foresti de Almeida-Toledo, Karine Frehner Kavalco

**Affiliations:** 1 Leiden University Medical Center, Department of Anatomy and Embryology, S-1-P, P.O. Box 9600, 2300 RC Leiden, The Netherlands; 2 Universidade Federal de Viçosa, Campus Rio Paranaíba, Institute of Biological and Health Sciences, Laboratory of Ecological and Evolutionary Genetics, BR 354 - km 310, PO Box 22, ZIP 38810-000, Rio Paranaíba, MG, Brazil; 3 Universidade Estadual do Sudoeste da Bahia, Campus Jequié, Department of Biological Sciences, Av. José Moreira Sobrinho s/n, Jequiezinho, ZIP 45206-190, Jequié, BA, Brazil; 4 Universidade de São Paulo. Institute of Biosciences, Department of Genetics and Evolutionary Biology, Rua do Matão, 277 – Edifício André Dreyfus, Cidade Universitária, ZIP 05508090, São Paulo, SP, Brazil

**Keywords:** Biodiversity, Catfish, Cytogenetics, Hypostominae, Teleostei

## Abstract

Several cytogenetic markers show chromosomal diversity in the fish such as “armoured catfish”. Although studies have characterized many species in the major genera representing these Siluridae, particularly in the genus *Hypostomus* Lacépède, 1803, trends in chromosome evolution of this group remain unclear. The Paraíba do Sul river basin contains the armoured catfish *Hypostomus affinis* Steindachner, 1877, which is unique because of its distribution of repetitive DNAs, the 5S and 18S rDNA. Identified samples and registered collections in Brazilian museums were identified as the same typological species, while we observed wide variations in the physical location of this gene in the karyotype based on fluorescent in situ hybridization results. In this study, we propose that these species can represent evolutionarily independent units, as these fish frequently undergo processes such as dispersion and vicariance and that the rDNA is associated with DNA that spreads in the genome, such as transposons. Additionally, the absence of gene flow due to the distance of the sample location could intensify evolutionary processes. The phenotypes found for the 18S rDNA showed minor changes in relation to the number of sites between the lower and upper drainage regions of Paraíba do Sul. The large difference in the number of sites found for the 5S rDNA entered the same region (upper drainage of the basin) and the literature data could represent a population dynamics where an expansion of the 5S rDNA sites provides an extinct or non-sampled cytotype in this work.

## Introduction

With a wide geographic distribution in nearly all of the Neotropical region from Costa Rica to Argentina, Loricariidae is considered one of the largest Neotropical fish families and the largest Family of catfishes (Siluriformes) (Nelson et al. 2016), with more than 1100 species described to date (Eschmeyer and Fong 2017).

The great diversity of armored catfish is also reflected in the available cytogenetic data of the group. Loricariidae exhibits large variations in diploid number, ranging from 2n = 36 chromosomes in *Loricaria latirostris* Boulenger, 1900 (Giuliano-Caetano 1998) to 2n = 96 in *Hemipsilichthys gobio* Lütken, 1874 (Kavalco et al. 2005, previously identified as *Upsilodus* sp.). This group shows several structural differences (Mariotto et al. 2009), numerous polymorphisms (Giuliano-Caetano 1998, Cereali et al. 2008), and morphologically differentiated sex chromosome systems (Alves et al. 2006, Oliveira et al. 2007, Konerat et al. 2015, Oliveira et al. 2015a, Rocha-Reis et al. unpublished data), which nearly always correspond to unique chromosomal features.

Most of this great diversity is related to the genus *Hypostomus* Lacépède, 1803, which contains approximately 200 valid species (Eschmeyer and Fong 2017), only some of which have their taxonomic resolution fully understood and resolved (Armbruster 2004). *Hypostomus* is considered one of the most diverse genus of Neotropical fish, and many genetic studies have examined their complex karyotype evolution (Rubert et al. 2008, Bitencourt et al. 2012, Endo et al. 2012, Pansonato-Alves et al. 2013, Traldi et al. 2013); studies have also been conducted to identify different species and detect phylogenetic relationships within the genus (Montoya-Burgos et al. 2002, Armbruster 2004, Lujan et al. 2015).

Fluorescent in situ hybridization (FISH) for localization of the 18S ribosomal RNA (18S rRNA) gene was one of the first cytogenetic-molecular markers applied in Neotropical fish (Hatanaka and Galetti Jr 2004), which revealed phenotypic variations in different groups. Although potentially interesting for gene expression studies, silver nitrate localization of Ag-NORs has not been widely used and is routinely applied only for comparison. Because not every 18S ribosomal DNA (18S rDNA) site is correctly identified using this technique (Dobigny et al. 2002), it is thought that the evolution of ribosomal genes can be determined from FISH data. These data, however, are rare for most fish, although some trends have been observed in smaller groups and have been examined in detail. In *Hypostomus*, through the efforts of different research groups, 18S gene localization data are available for approximately 30 species/populations (for review, see Rubert et al. 2016).

In contrast, data for 5S ribosomal DNA (5S rDNA) are limited. This marker has been defined in only approximately a dozen species of the genus for some Neotropical populations (Kavalco et al. 2004a, Mendes-Neto et al. 2011, Traldi et al. 2012, Pansonato-Alves et al. 2013, Traldi et al. 2013, Baumgärtner et al. 2014, Bueno et al. 2014, Rocha-Reis et al. unpublished data). Similar results were observed for the distribution of constitutive heterochromatin, although this type of highly compacted DNA requires further examination. The numerous chromosomes and their small sizes may be the main reason for the low prevalence of cytogenetic studies of armored catfish, despite their great species diversity and relative abundance in Brazilian rivers.

Although *Hypostomus affinis* Steindachner, 1877 was found in the Mucuri and Doce river basin, most of the records are related to the Paraíba do Sul river, indicating a wide distribution of this species in this river basin (Mazzoni et al. 1994). In this study, two populations of *H. affinis*, both upstream and downstream in the Paraíba do Sul River, were analyzed. Data for the evolution of ribosomal sequences were compared with polymorphisms observed in the populations presented here and those reported in the literature for the genus *Hypostomus*.

## Material and methods

**Figure 1.**
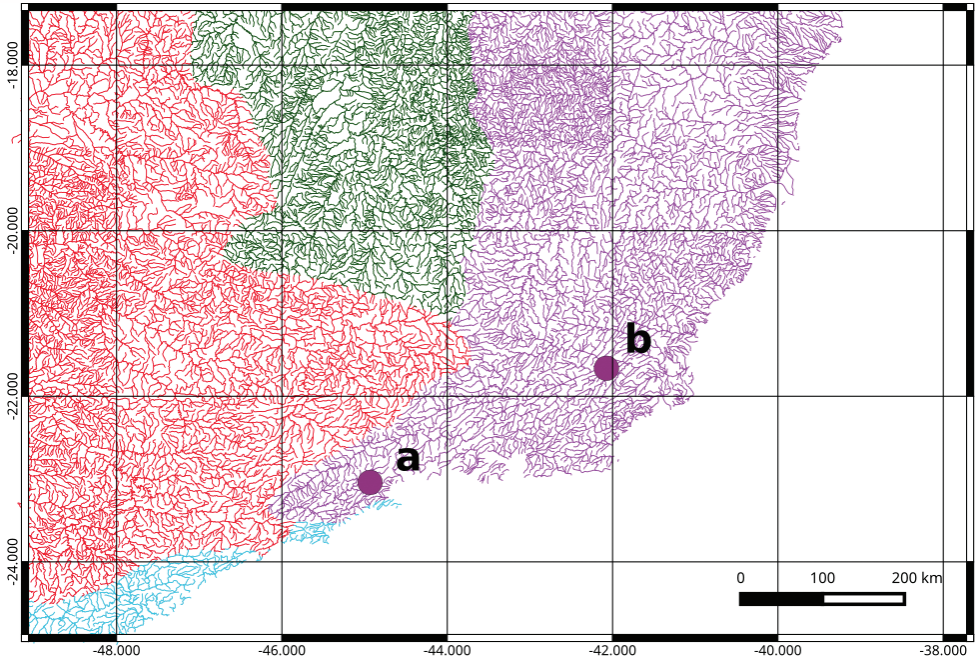
Hydrographic map of the southeast coast of Brazil with the collection points of *Hypostomus affinis*. Point “a” corresponds to Cunha/SP and point “b” corresponds to Itaocara/RJ. Hydrographic basins: Paraíba do Sul (in purple), São Francisco (in green), Upper Paraná (in red) and Rios Costeiros (in blue).

Two populations of *H. affinis* were collected from Jacuí creek, Cunha/SP (-23.04052/-44.93408, Fig. 1 – point a; one male/seven juvenile fish) and the Paraíba do Sul River, in Itaocara/RJ (-21.66141/-42.07454, Fig. 1 – point b; one female/five juvenile fish). Both collections were carried out in the year 2005. These samples were analyzed by classical and molecular cytogenetic techniques. First, the samples were processed, fixed in 10% formaldehyde, and stored in 70% ethanol. Finally, samples were sent to the Museum of Science and Technology of the Pontifical Catholic University of Rio Grande do Sul – MCP, where they were identified and deposited in the ichthyologic collection under vouchers MCP 43299 and MCP 43301 (populations from Cunha/SP and Itaocara/RJ, respectively).

The chromosomal preparations were obtained from kidney cells of the animals as described by Gold et al. (1990) with some modifications. Silver nitrate (Ag-NOR) staining to detect nuclear organizing regions (NORs) was performed according to Howell and Black (1980) and Kavalco and Pazza (2004), and C-banding followed a protocol adapted from Sumner (1972).

The physical location of the ribosomal genes was detected via FISH (Pinkel et al. 1986 modified by Pazza et al. 2006), using 18S ribosomal DNA (18S rDNA) and 5S ribosomal DNA (5S rDNA) probes obtained from the genome of *Prochilodus argenteus* Spix & Agassiz, 1829 (Hatanaka and Galetti Jr 2004) and *Megaleporinus elongatus* Valenciennes, 1850 (Martins and Galetti Jr 1999), respectively. The 18S and 5S rDNA probes were labeled with biotin-14-dATP by nick translation using BioNick Labeling System according to manufacturer instructions (Invitrogen).

Hybridization was detected with avidin and fluorescein isothiocyanate for 18S rDNA probes and Cy3 for 5S rDNA probes. Blade assembly was performed with antifade and propidium iodide, and antifade and DAPI for 18S rDNA and rDNA 5S probes, respectively. High-stringency washes with >75% (20% formamide/0.1× SSC) were performed for 15 min, and the signals were amplified using biotin-conjugated anti-avidin solution and incubated in non-fat dry milk buffer. Images were acquired with a camera coupled to an OLYMPUS BX41 microscope (Olympus Inc., Tokyo, Japan) using QCapture 6.0 (QImaging Surrey, BC, Canada) software.

To assemble the karyotypes, chromosomes were classified as metacentric (m), submetacentric (sm), subtelocentric (st), or acrocentric (a) according to the arm ratio proposed by Levan et al. (1964). All chromosomes were measured to avoid identification errors.

## Results

**Figure 2.**
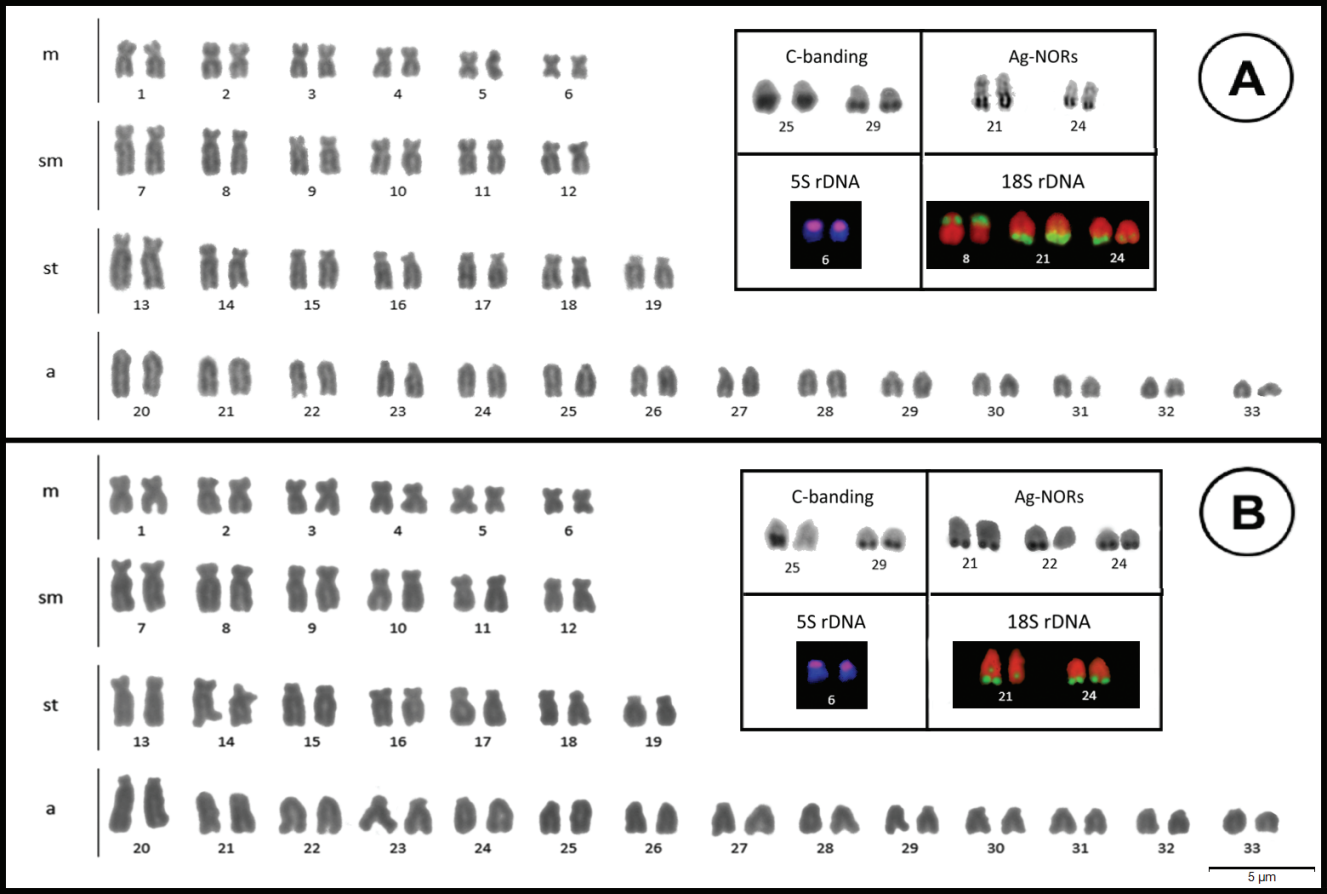
Karyotypes found for the populations of Cunha/SP (**A**) and Itaocara/RJ (**B**). In the boxes are the phenotypes for C banding, Ag-NORs and FISH with 5S and 18S rDNA probes.

Male and female fish in both populations showed a diploid number of 2n = 66 chromosomes, karyotype composed of 12m+12m+14st+28a, and fundamental number FN = 104 (Fig. 2A, B).

C-banding staining of both populations revealed subtle pericentromeric markers on several chromosomes, as well as conspicuous terminal blocks on two pairs of acrocentric chromosomes (a) (25, 29), although a size heteromorphism was found in one of the pairs in the population of Itaocara (25) (Fig. 2A, B, box). These markers did not correspond to the location of the major Ag-NORs (Fig. 2A, B, box).

Silver nitrate staining of both populations revealed the existence of multiple systems of NORs. In Cunha/SP specimens, two pairs of chromosome a (21, 24) exhibited large markers on their long arms (Fig. 2A, B). According to FISH, the 18S rDNA contained these four and two other sites located on the short arm of a small submetacentric (sm) pair, for a total of six gene sites (Fig. 2A, box). In Itaocara/RJ specimens, Ag-NORs analysis revealed five sites marked by silver nitrate: two located in the long arms of pairs a (21, 24) and another located in the terminal position of the short arm of chromosome a (22) (Fig. 2B, box). However, only four markers were detected on the 18S rDNA probe using FISH, corresponding to markers obtained from silver nitrate staining on chromosomes a (Fig. 2B, box).

Hybridization of the 5S rDNA probe revealed two sites marked in the lowest metacentric pair of the complement in both populations (pair 6) (Fig. 2A, B, box).

## Discussion

From a cytogenetic perspective, only one sample of *H. affinis* from the Jacuí creek Cunha/SP has been previously studied (Kavalco et al. 2004a, 2004b, 2005). In this study, we evaluated populations from the upper and lower Paraíba do Sul River. We first sought to expand sampling from the Jacuí creek to further analyze the heterochromatin polymorphism described previously (Kavalco et al. 2004b). Unexpectedly, we observed the conservation of chromosomal characteristics between the two populations analyzed in this study, as well as large variations, particularly with respect to the 5S rDNA sites compared to the previously described sample. Although geographically close, both populations from Cunha/SP showed large differences in their chromosomes. These populations showed relatively higher karyotypic divergence than geographic divergence, as they are only approximately 100 m away in geodesic distance and are part of the same drainage.

Although the chromosome number is the same and the karyotypic formula observed in the populations studied slightly differs from the previously sampled population, other cytogenetic features revealed differentiated evolutionary units. The difference in karyotype symmetry observed between the chromosomes of both samplings from Cunha/SP were clear; this was also clear when the relative size of the chromosomes was organized based on type, even when the same measurement and classification criterion proposed by Levan et al. (1964) and same magnification scale were used. In the previously analyzed sample from Jacuí creek, karyotypic asymmetry was clearly observed, even within each chromosomal group (Kavalco et al. 2005). In addition, the distribution of constitutive heterochromatin and existence of conspicuous blocks (Kavalco et al. 2004b) differed completely from the patterns observed in this study.

The difference among the observed chromosomal sites in the populations cannot be attributed to the use of 18S and 5S ribosomal DNA probes isolated from different species. The rRNA in eukaryotes presents as two subunits (one formed by 28S, 18S and 5.8S and another one formed by 5S) and their DNA sequences vary very slowly due to selective pressure, being considered highly conserved (Long and Dawid 1980). This allows the interspecific hybridization of the mentioned probes (obtained from *Prochilodus argenteus* and *Megaleporinus elongatus*), with chromosomes of a wide variety of fishes, like Characiformes (de Marco Ferro et al. 2001, Pazza et al. 2006, da Silva et al. 2016), Gymnotiformes (Fernandes et al. 2017a, 2017b) Perciformes (Jacobina et al. 2014, Argôlo and Affonso 2015, Oliveira et al. 2015b), Siluriformes (Blanco et al. 2014, Kantek et al. 2015, Ribeiro et al. 2015), including other species of *Hypostomus* (Kavalco et al 2004a, 2005, Traldi et al. 2013, Baumgärtner et al. 2014, Oliveira et al. 2015a, Lara Kamei et al. 2017).

For the location of 18S rDNA, we observed conservation of the number and position of sites in samples of the upper drainage region (Kavalco et al. 2005, this study), as well as chromosome differentiation in the lower Paraíba do Sul population, which showed the lowest number of sites. In addition to chromosome number, this is the only characteristic shared between samples from Jacuí creek.

The existence of different chromosomal formulas in close groups of different organisms, or nominally similar species, is attributed to chromosomal rearrangements. In armored catfish, two major types of chromosomal rearrangements appear to be involved in karyotype differences, depending on fixation of the diploid number (non-Robertsonian) or their variation (Robertsonian) (Artoni and Bertollo 1996, 2001, Kavalco et al. 2005). However, other factors should be considered in the chromosome evolution of the group, such as the dispersion trends of repetitive sequences such as ribosomal genes (Kavalco et al. 2004a). Because the presence of a pair of chromosomes carrying the rDNA in fish is thought to be a plesiomorphic condition (Martins and Galetti Jr 1999, Oliveira and Gosztonyi 2000), even for Loricariidae (Kavalco et al. 2004a, Alves et al. 2012), the genus *Hypostomus* may contain lines with contrasting tendencies (Pansonato-Alves et al. 2013) and possibly an ancestral phenotype with a site in a chromosomal pair (Traldi et al. 2013). Dispersion of ribosome cistrons may be related to not only species-specific events but also populational events, as in armored catfish in which the formation of isolated populations typically occurs because of low vagility (Artoni and Bertollo 2001, Bitencourt et al. 2012). In fact, variations in the distribution of 18S rDNA sites in the genus *Hypostomus* were clear, and it was difficult to establish evolutionary tendencies for the character, as observed among different populations of the Paraíba do Sul river. In addition, their co-location with DNAs similar to transposons (Pansonato-Alves et al. 2013) is unfavorable for observing macroevolutionary tendencies.

The divergent phenotype observed by Kavalco et al. (2004a) for the 5S rDNA cistrons in *Hypostomus* reflects well-known characteristics of genomic evolution in repetitive DNA. The evolutionary dynamics of this gene are related not only to variations in non-transcribed spacers, but also to synteny with long and short interspersed nuclear elements, non-long terminal repeat retrotransposons, U-snRNA families, and microsatellite polymorphisms (Rebordinos et al. 2013). According to these authors, polymorphisms in non-transcribed regions are observed in fish and polymorphisms in transcribed regions do not appear to interfere with the cellular activity of 5S rDNA. Although in some species, the molecular diversity of the 5S rDNA gene families is greater than the chromosome diversity (Rebordinos et al. 2013), this rule may not be applied for Neotropical ichthyofauna biodiversity. In several respects, the genus *Hypostomus*, as well as others, show various chromosomal evolutionary novelties at several levels, potentially reflecting recent adaptive radiation.

The speciation by allopatry can be an important source of diversity in Neotropics and could be responsible for the biodiversity of fishes from Brazilian rivers and it is possible that very short time periods can produce new phenotypes on *Hypostomus* chromosomes. At the same time, the extensive chromosomal variation observed in the sample of *H. affinis* analyzed previously by Kavalco et al. (2004a, 2004b, 2005; collected in the year 2001 - personal communication) could be related with an event that today represents a “dead end” in the evolutionary history of the population, highlighting sympatric evolutionary processes. Since the great number of 5S rDNA spread in the karyotype is an uncommon feature to the catfishes and it can increase chromosomal rearrangements, to consider the karyotype shown in this paper as the resident cytotype of the drainage is the most parsimonious idea. It is possible that the phenotype of the 18S rDNA disposition in the chromosomes shared between the individuals from Cunha/SP represents an evidence of introgression between a variant extinct cytotype and the ancient one, stated in this work. In this case, the variant form probably had lower adaptive value and was not able to fixation, or we do not sample the variant cytotype, just the ancient one.

## Conclusion

Minor chromosome changes were found between the two sampled populations, especially regarded to an extra chromosome pair bearing 18S rDNA in population from Cunha. In addition, 18S rDNA distribution in Cunha was the same as previously sample. However, the remarkable difference in the 5S rDNA distribution between two sampling at Cunha, separated by four years between the collections, could represent a population dynamic where an expansion of the 5S rDNA sites provide a phenotype furtherly extinct or not sampled in this work.
